# Comparison of physicians’ and dentists’ incident reports in open data from the Japan Council for Quality Health Care: a mixed-method study

**DOI:** 10.1186/s12903-023-02749-x

**Published:** 2023-02-02

**Authors:** Naomi Akiyama, Tomoya Akiyama, Hideaki Sato, Takeru Shiroiwa, Mitsuo Kishi

**Affiliations:** 1 School of Nursing, Gifu University of Health Science, 2-92 Higashi Uzura, Gifu City, Gifu Prefecture 500-8281 Japan; 2 Center for Postgraduate Clinical Training and Career Development, Nagoya University Hospital, 65 Tsurumai, Syowaku, Nagoya City, Aichi Prefecture 466-8560 Japan; 3 Department of Oral and Maxillofacial Surgery, Asahikawa Medical University, 2-1-1 Midorigaoka Higashi, Asahikawa City, Hokkaido 078-8510 Japan; 4 Center for Outcomes Research and Economic Evaluation for Health (C2H), National Institute of Public Health (NIPH), 2-3-6 Minami, Wako City, Saitama Prefecture 351-0197 Japan; 5 School of Dentistry, Iwate Medical University, 19-1 Uchimaru, Morioka City, Iwate Prefecture 020-8505 Japan

**Keywords:** Dentistry, Adverse event, Incident report, Error, Intern, Patient safety, Content analysis, SHELL model, Mixed-methods study

## Abstract

**Background:**

Patient safety is associated with patient outcomes. However, there is insufficient evidence of patient safety in the dental field. This study aimed to compare incidents reported by dentists and physicians, compare the type of errors made by them, and identify how dentists prevent dental errors.

**Methods:**

A mixed-methods study was conducted using open data from the Japan Council for Quality Health Care database. A total of 6071 incident reports submitted for the period 2016–2020 were analyzed; the number of dentists’ incident reports was 144, and the number of physicians’ incident reports was 5927.

**Results:**

The percentage of dental intern reporters was higher than that of medical intern reporters (dentists: n = 12, 8.3%; physicians: n = 180, 3.0%;* p* = 0.002). The percentage of reports by dentists was greater than that by physicians: wrong part of body treated (dentists: n = 26, 18.1%; physicians: n = 120, 2.0%; * p* < 0.001), leaving foreign matter in the body (dentists: n = 15, 10.4%; physicians: n = 182, 3.1%; * p* < 0.001), and accidental ingestion (dentists: n = 8, 5.6%; physicians: n = 8, 0.1%; * p* < 0.001), and aspiration of foreign body (dentists: n = 5, 3.4%; physicians: n = 33, 0.6%; * p* = 0.002). The percentage of each type of prevention method utilized was as follows: software 27.8% (n = 292), hardware (e.g., developing a new system) 2.1% (n = 22), environment (e.g., coordinating the activities of staff) 4.2% (n = 44), liveware (e.g., reviewing procedure, double checking, evaluating judgement calls made) 51.6% (n = 542), and liveware-liveware (e.g., developing adequate treatment plans, conducting appropriate postoperative evaluations, selecting appropriate equipment and adequately trained medical staff) 14.3% (n = 150).

**Conclusion:**

Hardware and software and environment components accounted for a small percentage of the errors made, while the components of liveware and liveware-liveware errors were larger. Human error cannot be prevented by individual efforts alone; thus, a systematic and holistic approach needs to be developed by the medical community.

## Background

If medical error was a disease, it would rank as the third leading cause of death in the US [1]. Medical errors affect patients’ quality of life and can increase the economic burden on society [2]. Medical accidents, in which patient injuries result from medical care, affect patients in various ways and may disrupt entire healthcare organizations. Managing patient safety is one of the elements necessary to advance the development of healthcare organizations.

Ensaldo-Carrasco et al. reviewed 40 articles on ambulatory dental care, findings errors were reported, such as in diagnosis and examination, treatment planning, communication procedural errors, and the accidental ingestion or inhalation of foreign objects [3]. Choking can represent a serious adverse event for patients, potentially leading to mortality or permanent impairment [4]. Errors arising from the failure to acquire informed consent from a patient, the failure to establish and maintain appropriate infection control measures, and the failure to formulate a proper diagnosis can lead to negative consequences [5]. In particular, with the increasing number of elderly patients, the prescription of medication has become a complicated task that may lead to errors [5, 6]. Currently, general dental practices are carrying out more involved dental procedures and surgeries at their consulting rooms [7]. Although dental practitioners generally provide complex medical care, little research has been conducted on the safety of dental inpatients [5–8].

Mortality and significant morbidity associated with the practice of medicine have led to many strategies being developed to help improve patient safety; however, due to its lack of associated mortality and lower associated morbidity, dentistry has been slower at systematically considering how patient safety can be improved [9]. From a patient safety perspective, a number of peculiarities associated with dentistry serve to distinguish it from other areas of healthcare, particularly from healthcare that is administered within hospitals. To improve quality and safety in dentistry, it is necessary to analyze and understand the characteristics of dentistry-specific incidents and to take appropriate measures and educate dental professionals [10]. Dental care is usually less aggressive than treatment received in hospital and consequently results in comparatively less harm [11]. Individuals, teams, and systems must work together in the appropriate environment to ensure safety and quality care for patients [12].

## Methods

### Aim

Identifying and recording the occurrence of an incident is the first step in the reporting and learning process [13]. This study quantitatively compared incident reporting by dentists and physicians and the types of errors made. The qualitative objective was to identify what dentists did to prevent errors.

### Design

A mixed-methods study was conducted using publicly available data obtained from the Japan Council for Quality Health Care (JCQHC).

### Data collection

This study was conducted using data from the JCQHC. The JCQHC has been collecting incident reports from all over Japan since 2014. The incidents that were analyzed had been reported by 1512 hospitals in the year 2019. The JCQHC website includes freely available data with respect to reported medical incidents and these data have been the subject of research in previous studies. For example, Akiyama et al. utilized the JCQHC data to investigate incident reports involving hospital administrative staff [14]. Ichikawa et al. researched physicians’ emotions and how this influenced the occurrence of medical errors [15]. This study used the data from the JCQHC, which includes reports of incidents by physicians and dentists for the period January 2017–2019. The data were downloaded from the JCQHC website on August 14, 2020. A total of 6071 incident reports were collected, of which 144 incidents had been reported by dentists and 5927 which had been reported by physicians; we used all data in the analysis. Incident reports contain two types of data: quantitative and contextual. Quantitative data include the characteristics of the incident reports, when/where incident occurred, who was involved, and the number of staff were involved. Contextual data include a summary of the incident, details of the incident, what treatment followed the incident, and how preventive policies were subsequently generated. These data were generated by the reporter of the incident.

### Analysis

#### Quantitative data analysis

The JCQHC data contains the time the incident occurred, the place, report content, additional medical care needed following the error, the year the incident was reported, and the type of error. These data were treated as categorical. The reporter category was divided using the length of experience data of the JCQHC into medical interns (less than one year of experience) and others (one year or more of experience). We compared the percentage of incident reports of physicians and dentists using the χ^2^ test and Fisher’s exact test. The statistical analysis was performed using JMP statistical software (version 12.0); and statistical significance was set at * p* < 0.05.

#### Contextual data analysis

For analysis of contextual data, we focused on whether there were policies in place in the dental care field to prevent similar errors from occurring in the future, which we analyzed using content analysis. Content analysis is useful for examining trends and patterns in documents [16]. Two registered nurses with experience in content analysis research and the field of hospital management, assisted in analyzing the data. One of the nurses had experience as a patient safety administrator in a hospital and the other had experience as a hospital administrator. One of the researchers read the data to gain an overall understanding of the content related to the prevention of similar errors and divided the text into units of meaning, after which another researcher carried out the process of division into units of meaning again; the two researchers discussed this division. The researchers then continued discussion on any units that still presented ambiguity or abstraction, until consensus was achieved. Discussions continued until of all the researchers agreed with the results of the study.

The meaning units were organized under the SHELL model, which was developed and advocated principally in aviation literature by Hawkins and Orlady [17]. These components include software (procedure, protocol, and training), hardware (machines, medical equipment and instruments), environment (operating theatre, wards, and consultation rooms), and liveware (doctors, nurses, and other healthcare professionals, as well as patients). Liveware involves interrelationships among individuals within and between groups and can be organized in two ways: first, as the person concerned, and second, as those people around the person concerned. We categorized the person concerned as “liveware” and the persons around the person concerned as “liveware-liveware.”

## Results

### Characteristics of the incidents reported

The characteristics of the incidents reported to the JCQHC are listed in Table 1. The number of incidents reported by dentists to have “occurred on a weekday” was higher than the number of incidents reported by physicians (dentists: n = 140, 97.2%; physicians: n = 5324, 89.8%; * p* = 0.002). We found that dentists reported that the most common location for an incident to occur was “outpatient department” (dentists: n = 69, 47.9%; physicians: n = 847, 14.3%; * p* < 0.001). The percentage of report contents differed between dentists and physicians (* p* < 0.001); the highest percentage of incidents reported by dentists took place during “treatment” (n = 104, 72.2%), which was also the case for physicians (n = 3215, 54.2%). The percentage of incidents reported by dental interns was higher than that of medical interns (dentists: n = 12, 8.3%; physicians: n = 180, 3.0%; * p* = 0.002). However, there were no differences in the percentage of reported additional medical care needed as a result of errors made between dentists and physicians.

**Table 1:** Characteristics of the incidents reported

	Dentists n = 144 n (%)	Physicians n = 5927 n (%)	* p* value
*Occurring time*			
Weekday	140 (97.2)	5324 (89.8)	0.002^a^
Weekend	4 (2.8)	603 (10.2)	
*Occurring place*			
Inpatient department	75 (52.1)	5080 (85.7)	< 0.001^b^
Outpatient department	69 (47.9)	847 (14.3)	
*Report contents*			
Treatment	104 (72.2)	3215 (54.2)	< 0.001^a^
Medical devices	11 (7.6)	146 (2.5)	
Medicine	3 (2.1)	584 (9.9)	
Examination	3 (2.1)	557 (9.4)	
Drain, tubes	3 (2.1)	388 (6.5)	
Others	20 (13.9)	1037 (17.5)	
*Reporter*			
Medical intern	12 (8.3)	180 (3.0)	0.002^a^
Others	132 (91.7)	5747 (97.0)	
*Additional medical care attributed to errors*			
Needed	127 (88.2)	5377 (90.7)	0.308^b^
Not needed	17 (11.8)	550 (9.3)	
*Incident reporting year*			
2017	40 (27.8)	1762 (29.7)	0.233^b^
2018	45 (31.3)	2135 (36.0)	
2019	59 (41.0)	2030 (34.3)	

^a^Fisher’s exact test

^b^χ^2^ test

### Types of errors reported

The percentages of all types of errors made are recorded in Table 2. The percentage of errors reported by dentists relating to “wrong part of body treated” was higher than that of physicians (dentists: n = 26, 18.1%; physicians: n = 120, 2.0%; * p* < 0.001). Dentists were also more likely “leaving foreign matter in the body” (dentists: n = 15, 10.4%; physicians: n = 182, 3.1%; * p* < 0.001). The “accidental ingestion” percentage was higher among dentists than physicians (dentists: n = 8, 5.6%; physicians: n = 8, 0.1%; * p* < 0.001), and “aspiration of foreign body” was also more frequently reported among dentists (dentists: n = 5, 3.4%; physicians: n = 33, 0.6%; * p* = 0.002).

**Table 2:** Type of errors reported

	Dentist n = 144 n (%)	Physicians n = 5927 n (%)	* p* value
Wrong treatment	34 (23.6)	2318 (39.1)	< 0.001
Wrong part of body treated	26 (18.1)	120 (2.0)	< 0.001
Leaving foreign matter in the body	15 (10.4)	182 (3.1)	< 0.001
Wrong treatment methods	8 (5.6)	447 (7.6)	0.520
Accidental ingestion	8 (5.6)	8 (0.1)	< 0.001
Aspiration of foreign body	5 (3.4)	33 (0.6)	0.002
Patient misidentification	1 (0.7)	25 (0.4)	0.465
Others	47 (32.6)	2794 (47.1)	< 0.001

Using χ^2^ test

### Methods utilized to prevent further incidents from occurring

The methods that dentists used to try to prevent further incidents from occurring are shown in Table 3. These methods are related to software, hardware, environment, liveware, and liveware-liveware. Table 3 does not include two out of 144 cases (1.4%) involving patient-oriented diseases; these cases were excluded from our analysis and marked as “non-preventable accidents.”

In terms of software, we found nine applicable prevention methods, and we examined the top three categories: formulating a manual/rule (n = 108, 37.0%), training/education (n = 96, 32.9%), and attending conferences (n = 28, 9.6%). Concerning hardware, there was only one incident reported and one category defined, developing a new system (n = 22, 100.0%). With regard to environment, there were three categories developed: coordinating the activities of staff (n = 21, 47.7%), improving the physical environment (n = 14, 31.8%), and rearranging the schedule (n = 9, 20.5%). For liveware, we described the five most frequent categories as follows: review of procedure (n = 104, 19.2%), double checking (n = 100, 18.5%), evaluating judgement calls made (n = 94, 17.3%), sharing of information (n = 54, 10.0%), and compliance with rules (n = 49, 9.0%). For liveware-liveware, there were seven categories developed and we examined the most frequent of these: formulating an adequate treatment plan (n = 61, 40.7%), appropriate postoperative evaluation (n = 34, 22.7%), and selecting appropriate equipment or adequately trained medical staff (n = 26, 17.3%).

The percentage of each category of method utilized to prevent further incidents was as follows: software 27.8% (n = 292), hardware 2.1% (n = 22), environment 4.2% (n = 44), liveware 51.6% (n = 542), and liveware-liveware 14.3% (n = 150).

**Table 3:** Methods utilized by dentists to prevent further incidents from occurring

		n (%)
Software		292 (100.0)
	Formulating a manual and/or rule	108 (37.0)
	Training and/or education	96 (32.9)
	Attending conferences	28 (9.6)
	Doing timeout	19 (6.5)
	Developing a culture of safety	16 (5.5)
	Patient engagement	11 (3.8)
	Report the accident	10 (3.4)
	Patient education	3 (1.0)
	Stopping or postponing the operation	1 (0.3)
Hardware		22 (100.0)
	Developing a new system	22 (100.0)
Environment		44 (100.0)
	Coordinating the activities of staff (including the lack of experienced staff, instructors)	21 (47.7)
	Improving the physical environment	14 (31.8)
	Rearranging the schedule	9 (20.5)
Liveware		542 (100.0)
	Review of the procedure	104 (19.2)
	Double checking	100 (18.5)
	Evaluating judgement calls made	94 (17.3)
	Sharing of information	54 (10.0)
	Compliance with the rules	49 (9.0)
	Verifying observations	35 (6.5)
	Double checking	29 (5.4)
	Creating a medical record	19 (3.5)
	Providing information to the patient	19 (3.5)
	Paying attention to the patient	18 (3.3)
	Verbal checking	9 (1.7)
	Selecting appropriate medication	5 (0.9)
	Directly consulting senior dentists	4 (0.7)
	Using appropriate dosage of medication	3 (0.6)
Liveware-Liveware		150 (100.0)
	Formulating an adequate treatment plan	61 (40.7)
	Appropriate postoperative evaluation	34 (22.7)
	Selecting appropriate equipment or adequately trained medical staff	26 (17.3)
	Checking treatment equipment before usage	9 (6.0)
	Checking equipment during and after use	8 (5.3)
	Appropriate perioperative management	8 (5.3)
	Preparation for emergency response	4 (2.7)

## Discussion

In this study, we compared dentists’ and physicians’ incident reporting and the types of errors made and identified what dentists did to prevent errors. Dentists’ reported incidents predominately occurred on weekdays and in the outpatient department during treatment. Of our many findings, we focused on three major results: (1) the percentage of interns who reported incidents was higher among dentists than physicians, (2) there was a difference in the types of incidents reported by dentists and physicians, and (3) reporter-focused prevention methods bias.

### Dental interns were more likely to report incidents than were medical interns

Our study revealed that dental interns’ reporting percentages were higher than those of physicians’. Sakuma et al. reported that resident trainee dentists experienced more incidents of a higher level of severity than did staff dentists in Japan [18]. Almost half of the participants who were intern doctors had been assigned to tasks for which they were not trained or for which medical errors could have happened easily [19]. Medical interns are not only factors in the liveware category, which included missed treatment procedures or missed judgements, but also the environmental category, which included the lack of experienced staff and instructors. Trainee physicians reporting negative workplace conditions are more likely to report burnout/stress [20]. Hospitals are obliged to develop new methods to prevent further incidents from occurring and thereby protecting dental interns from preventable errors.

### Difference in types of incidents reported by dentists and physicians

The characteristics of the incidents reported by dentists related to incorrectness; for example, when the incorrect body part was treated, or a wrong part of body treated was provided. The data analyzed in our study were collected from hospitals, and the patients in this study were likely to have been referred to the hospital by a local dental clinic. Studies have shown that diligence and attentiveness falter when a patient is referred to or transferred to another healthcare provider [21]. In the case of referrals from a clinic to the hospital, the transition will be paper-based, and the patient will utilize a letter of referral. The dentition of the patient is sometimes poor. Referring only to the referral letter may lead to adverse outcomes. To prevent the incorrect body part receiving treatment, it is necessary for patients and their families to be aware of the problem for which they are being referred. It is also worthwhile for the patient to bring specific data and information, such as X-rays, when they are referred to another medical practitioner. It is difficult to directly connect local and hospital dentists because these two types of dentist use different local electronic systems rather than a global network. However, in the future, it would be beneficial to develop a new system, so that local dentists could send patient reports and related documentation, such as X-rays, through a secure information channel. Then, the hospital dentist could confirm the tooth/teeth that require diagnosis and treatment.

### Reporters focused on human factors, not on software, hardware, or the environment

We identified threats to patient safety when analyzing the dental incident reports as it appeared that the reporter was biased and focused on human methods of prevention as a personal approach. The liveware components of the SHELL model comprised a high percentage of the prevention methods. Focusing on the individual origins of error isolates unsafe acts from their system context; finally, human error tends to be overlooked [22]. Our research revealed that prevention methods such as “attending conferences” and “organization of staff”—software and environment components of the SHELL model, respectively—comprised a small percentage of all methods. Reis et al. reported that the dimensions of patient safety that proved strongest were “teamwork within units,” while proven weaknesses were “staffing,” “handoffs management and transitions,” and “lack of teamwork across units” [23].

We found that the reporters focused on the individual regarding prevention methods, such as observation, appropriate selection of procedures, and judgement. To prevent choking injuries, including accidental ingestion and aspiration of foreign body, requires adequate risk perception ability [4]. Our research also revealed that the fundamental solution is to improve the ability of medical staff to predict risks. Risk perception training and education should be improved in the hospital context. This represents a systems approach that could assist in the development of a culture of safety.

In Japan, “the administrator of hospital, clinic, or birthing center shall undertake measures to ensure safety in medical care in said hospital, clinic, to the provisions of an Ordinance of the Ministry of Health, Labour and Welfare”; this law is defined under the Medical Care Act [24]. The Enforcement Regulations of the Medical Care Act were amended in 2016 to note that the administrator of an advanced medical treatment facility must assign a particular individual who is responsible for medical safety management and must establish a department of safety management related to medical care that employs full-time physicians, pharmacists, and nurses [25]. According to a survey related to the staffing of medical safety management departments that was conducted among 51 national university hospitals, there were no full-time dentists in the patient safety management department [26]. Our results indicate that the incidents reported by physicians are different, and it is necessary to formulate recurrence prevention measures using a systems approach by medical professionals who are familiar with the dental field to foster a culture of medical safety. We propose that the patient safety departments in medical facilities should collaborate with dental professionals, such as dentists and dental hygienists, especially when considering preventive measures related to hard/software and the environment; these require specific information obtainable only from dental professionals’ files to implement a holistic preventive strategy.

## Limitations

Our findings present the characteristics of incidents reported by dentists and trends in prevention methods in the dental field. However, this study had several limitations. First, the study utilized secondary data from the JCQHC website. The textual data in our study were limited to incident reporters’ descriptions. Due to such a limitation, our findings on the interpretation of methods of prevention are limited, as we were not able to include the background information of hospitals or their staff. In this study, content analysis was conducted by two experienced researchers, but independent coding and calculation of interrater agreement would be beneficial for future research. Differences in trends in the frequency of incidents between dentists and physicians, as obtained in this study, may be discussed, but we used JCQHC public data and did not calculate the frequency of occurrence of incidents in routine clinical practice. Second, the lack of prior research on incidents reported by dentists made it difficult to establish whether the prevention methods shown in our findings were sufficient. Third, the sample size of our study was small. There was a discrepancy in the number of incidents reported by physicians versus the number of incidents reported by dentists. Although the number of incidents reported by dentists was small, dental interns reported a higher percentage of incidents than did physicians’ interns. The low number of incidents reported by dentists may be because dentists are less likely to experience as many incidents as physicians, but as our data were collected from a secondary source, we cannot suggest this as a finding and recommend that further research be conducted.

## Conclusion

Our findings revealed that dental interns reported a higher percentage of incidents than did medical interns. We suggest that hospital administrators and educators need to focus more attention on the systems surrounding dental interns to assist them in error prevention. Dental interns need to be trained well in procedures that support a culture of safety, and be supported by veteran physicians and dentists whose supervision would contribute to the prevention or at least minimization of these errors. In addition, we found an imbalance in the prevention methods utilized by dentists. The hard/software and environment components accounted for a small percentage of the errors made, while the components of liveware and liveware-liveware errors were larger. Human error cannot be prevented by individual efforts alone; thus, a systematic and holistic approach needs to be developed by the medical community.

## Data Availability

The datasets generated during and/or analyzed during the current study are available from the corresponding author on reasonable request.

## Abbreviations

JCQHC: Japan Council for Quality Health Care
